# Optimal Indocyanine Green Dosage for Repetitive Angiography for Laparoscopic Colorectal Surgery

**DOI:** 10.3390/medicina60121966

**Published:** 2024-11-29

**Authors:** Gyung Mo Son, Sang-Ho Park, Nam Su Kim, Mi Sook Yun, In Young Lee, Myeong-Sook Kwon, Tae Kyun Kim, Eun Hwa Lee, Eun Jung Hwang, Kwang-Ryul Baek

**Affiliations:** 1 Department of Surgery, Pusan National University Yangsan Hospital, Pusan National University School of Medicine, Yangsan 50612, Republic of Korea; 2 Research Institute for Convergence of Biomedical Science and Technology, Pusan National University Yangsan Hospital, Yangsan 50612, Republic of Korea; msyun@pusan.ac.kr (M.S.Y.); vmffkdl38@naver.com (I.Y.L.); 1506audtnr@hanmail.net (M.-S.K.); 3 Department of Electronic Engineering, Pusan National University, Busan 46241, Republic of Korea; propia@pusan.ac.kr (S.-H.P.); namsu7335@naver.com (N.S.K.); krbaek@pusan.ac.kr (K.-R.B.); 4 Department of Anesthesia and Pain Medicine, Pusan National University School of Medicine, Busan 50612, Republic of Korea; anesktk@pusan.ac.kr; 5 Department of Pharmacy, Pusan National University Yangsan Hospital, Yangsan 50612, Republic of Korea; eunhwalee123@gmail.com (E.H.L.); ejhwang@pusan.ac.kr (E.J.H.)

**Keywords:** indocyanine green, angiography, colorectal surgery, laparoscopy, near-infrared, perfusion imaging, artificial intelligence

## Abstract

*Background and Objectives*: This study aimed to determine the minimal effective dose of indocyanine green (ICG) required for accurately assessing colonic perfusion during laparoscopic colorectal surgery using a laser-assisted laparoscopic near-infrared (NIR) camera system. *Materials and Methods*: In 15 patients with colorectal cancer undergoing right hemicolectomy, the left branch of the middle colic artery was preserved, and ICG angiography was performed in the transverse colon. To determine the optimal ICG dose, experimental doses of 0.01, 0.02, 0.03, 0.04, and 0.05 mg of ICG per patient’s body weight (kg) were administered intravenously in each group. Additionally, a conventional dose of 0.2 mg/kg was administered in the same patients more than 30 min after the initial dose. For quantitative analysis, the fluorescent expression region was extracted, and fluorescence intensity was analyzed using automatic image processing. Analysis accessibility, T_1/2MAX_, perfusion time ratio, slope, artificial intelligence (AI)-based perfusion pattern analysis, and washout time were measured in 150 detailed regions of interest in each image. *Results*: Group 1 (0.01 mg/kg) showed significantly lower accessibility rates for quantitative analysis (48.0%) compared with Groups 2–5 (84.7–100%). The mean slope value in Group 1 was 3.7, which fell below the acceptable threshold (>4) and was significantly lower than that of the other groups (*p* < 0.001). An acceptable AI-based perfusion pattern was 14.2% in Group 1, significantly lower than in Groups 2–5 (66.4–100%). Washout time was significantly faster with minimal doses compared with conventional doses (39.0 ± 15.8 s vs. 117.5 ± 4.9 s, respectively, *p* < 0.001). *Conclusions*: This study supports the use of minimal ICG doses, ranging from 0.02 to 0.05 mg/kg, to optimize repetitive ICG angiography using a laser-assisted laparoscopic NIR camera.

## 1. Introduction

Minimally invasive surgery has become increasingly favored for treating colorectal cancer [1,2]. A key aspect of oncologic radical surgery is the high ligation of the feeding vessels at their origin from major vascular structures, enabling radical lymph node dissection [3,4]; however, approximately 10% of patients have an underdeveloped marginal arcade for collateral circulation in the colon mesentery, particularly around the splenic flexure [5]. In such cases, reduced blood flow can lead to ischemia in the remaining colon tissue. If this ischemia is not detected prior to anastomosis, it can lead to severe complications, including anastomotic leakage, colonic necrosis, and sepsis [6,7,8].

Traditionally, intraoperative evaluation of colonic perfusion has relied on subjective methods such as visual inspection of the colon wall color or peristalsis and assessment of vascular pulsation. These subjective methods have low accuracy of perfusion status assessment [9,10].

Recent advancements in near-infrared (NIR) imaging technology have introduced the use of indocyanine green (ICG) angiography in conjunction with laparoscopic NIR cameras to assess blood perfusion more reliably [11,12,13,14,15,16]. ICG angiography is a simple and cost-effective method for evaluating colonic perfusion during colorectal surgery. ICG, which was approved by the U.S. Food and Drug Administration in 1959, is widely used for diagnostic purposes, including evaluating liver and cardiac function. In laparoscopic colon surgery, ICG is typically administered at doses of 0.2–0.25 mg/kg to assess colonic perfusion [9,10]. Newer laparoscopic NIR cameras, particularly those with single-wavelength lasers, provide improved fluorescence intensity (FI) and image resolution compared with conventional NIR camera systems that use broad-spectrum xenon lamps [16,17,18].

Recent studies have questioned the conventional ICG dosing protocol [19,20]. Kidney transplantation to assess renal graft blood flow found that a lower dose of 0.02 mg/kg provided more accurate results than the conventional 0.2 mg/kg dose study using a laser-assisted laparoscopic NIR camera [20].

This study aimed to determine the minimal ICG dose required for quantitative perfusion analysis during laparoscopic colorectal surgery using a laser-assisted laparoscopic NIR camera system. Establishing this minimal dose could contribute to standardizing ICG angiography in colorectal surgery and improve both surgical precision and patient safety.

## 2. Materials and Methods

### 2.1. Patients

This prospective experimental study was conducted at Pusan National University Yangsan Hospital (PNUYH) from April 2019 to January 2020. It included patients with right-sided colon cancer who underwent laparoscopic right hemicolectomy. The inclusion criteria consisted of patients aged 19 to 80 years, classified as American Society of Anesthesiologists (ASA) Grade 1 or 2 (Grade 1: healthy patients; Grade 2: patients with mild systemic disease). Exclusion criteria included patients with distant metastasis, hemodynamic instability, those undergoing emergency surgery, and patients with allergies to radiologic contrast agents or side effects related to sodium iodide. All participants provided written informed consent voluntarily after receiving approval from the PNUYH Institutional Review Board (IRB No. 05-2019-024) and Korea’s Ministry of Food and Drug Safety (KMFDS IRB No. 32096).

### 2.2. Sample Size Calculation

A perfusion time ratio (TR) value below 0.60, as one of the quantitative perfusion parameters, indicates good perfusion status, falling within an acceptable range and associated with safe anastomosis in a previous study [9]. The mean and standard deviation of TR for favorable perfusion status were 0.38 and 0.10, respectively. Poor perfusion status was anticipated to show statistical differences outside the 95% confidence interval for TR. A significance level of 5%, power of 80%, expected difference of 0.21, standard deviation of 0.10, and dropout rate of 10% were considered. The required sample size was calculated to be 2.18 patients per minimal dose group (0.01–0.05 mg/kg) to compare the conventional dose (0.2 mg/kg). A total of 15 patients were planned for inclusion in this study, with three patients assigned sequentially to one of five minimal dose groups. Each group received an initial experimental ICG dose (0.01–0.05 mg/kg), followed by a second conventional dose (0.2 mg/kg) administered more than 30 min after the initial dose (Table 1).

### 2.3. ICG Dosage Protocol

**Figure 1:**
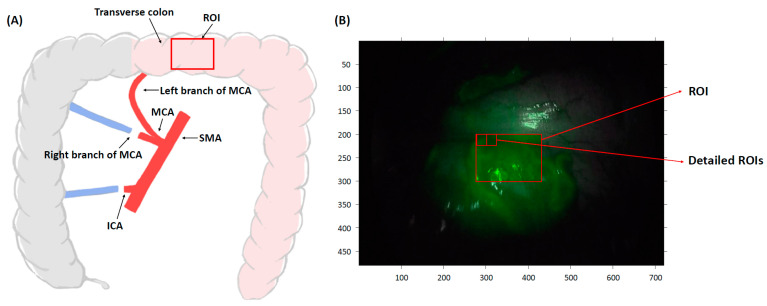
Schematic diagram of laparoscopic right hemicolectomy. (**A**) Preservation of the left branch of MCA ensures adequate blood flow to the transverse colon, resulting in favorable perfusion conditions. For quantitative perfusion analysis, the ROI was set at a transverse colon segment on the fluorescence image. (**B**) The ROI is defined as a central area with a width of 154 pixels and a height of 101 pixels. To reduce motion artifact-related errors, a detailed ROI of 10 × 10 pixels was used. SMA; superior mesenteric artery, MCA; middle colic artery, ICA; ileocolic artery, ROI; region of interest.

Preservation of the left branch of the middle colic artery (MCA) ensures continuous and adequate blood flow to the transverse colon, thus maintaining appropriate perfusion conditions after the right hemicolectomy (Figure 1A). Thus, quantitative perfusion parameters should reflect a favorable perfusion status. In all patients, the left branch of the MCA was preserved during surgery, and favorable colonic perfusion with adequate blood flow was confirmed by observing backflow from the mesenteric marginal artery during mesentery division.

ICG (Diagnogreen, 25 mg, Daiichi Sankyo, Tokyo, Japan) was diluted in 10 mL of distilled water and slowly injected into the patient’s brachial vein by an anesthesiologist at the surgeon’s request. The experimental minimum doses of ICG (0.01–0.05 mg/kg for each group) were administered according to the patient’s body weight. As a control, a conventional dose of 0.2 mg/kg was readministered to the same patient more than 30 min after the initial dose, prior to the completion of surgery. Following the ICG injection, NIR light at 805 nm was emitted, and the central portion of the transverse colon, within the MCA territory that receives blood flow from the left branch of the MCA, was imaged for 2 min using a laser-assisted laparoscopic NIR camera (1588 AIM camera system, Stryker, Kalamazoo, MI, USA). The region of interest (ROI) was defined as a region with a width of 154 pixels and a height of 101 pixels centered in the image. A smaller ROI of 10 pixels in width and 10 pixels in height was defined as the detailed ROI (Figure 1B). The FI changes over time were determined in the ROIs. A repeated analysis was performed by setting 150 detailed ROIs in each operation recording video.

### 2.4. Fluorescence Image Processing

A laparoscopic NIR camera utilizing laser technology was employed to enhance the visualization of fluorescence images. The camera captures two monotone images: one in the visible light wavelength range ($I_{nir}$) and one in the NIR wavelength range corresponding to ICG ($I_{vis}$). The visible light image ($I_{vis}$) is displayed in grayscale, while the NIR image ($I_{nir}$) is displayed in green scale. These two images are then combined to form a synthesized image ($I_{mix}$), which is displayed as the final output.

$$I_{mix}=\left[I_{vis},\;I_{vis}+I_{nir},\;I_{vis}\right]$$

In the synthesized image ($I_{mix}$), the matrix represents the red, green, and blue (RGB) color channels, with each channel corresponding to specific components of the visible and NIR images.

Consequently, the absolute value of the green (G) channel can increase if the brightness of the grayscale image is affected by backlighting or other fluorescence sources, even when the detected FI remains constant. The formula to isolate the fluorescence component from the RGB image is given by:

$$I_{nir}\left(i,\;j\right)=I_{mix}\left(i,\;j,\;2\right)\;-\frac{\left(I_{mix}\left(i,\;j,\;1\right)\;+\;I_{mix}\left(i,\;j,\;3\right)\right)}{2}$$

**Figure 2:**
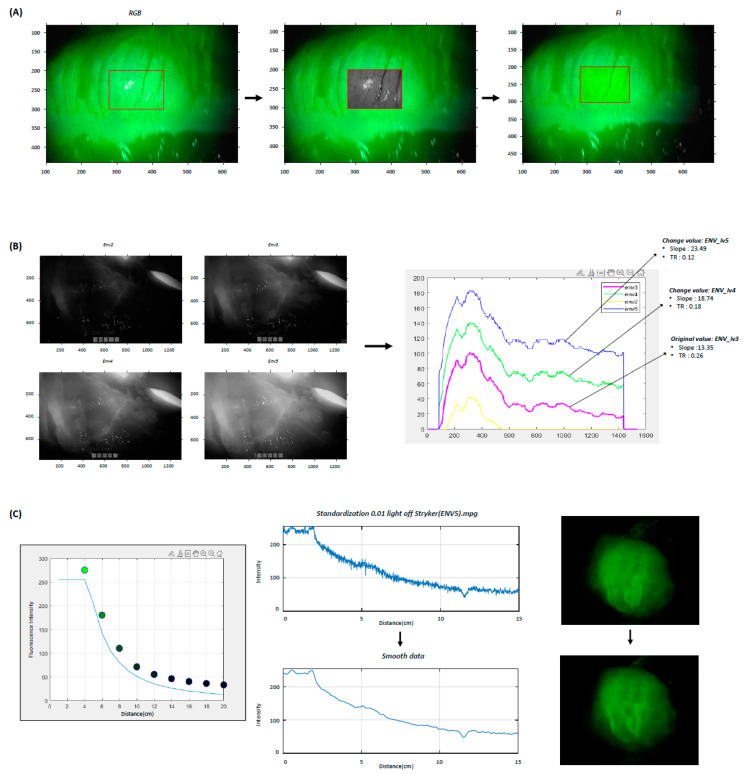
Fluorescence Image Processing. (**A**) The original image from the RGB channel contains a combination of the black-and-white image and the fluorescence signal within the ROI (red box). The fluorescence signal is extracted exclusively from this combination image, and the grayscale component is subtracted. After grayscale removal, the fluorescence signal within the ROI is obtained. (**B**) ENV modes, ranging from 0 to 5, provide different FI. The ENV level influences the shape and position of the FI contrast curve. Image processing is applied to transform the ENV level in fluorescence images. (**C**) The received fluorescence signal is inversely proportional to the square of the distance from the camera. To compensate for this noise, a Gaussian moving average filter is applied to obtain a clear graph pattern. RGB; the red, green, and blue color channels, FI; fluorescence intensity, ROI; region of interest, ENV; endoscopic near-infrared visualization, TR; perfusion time ratio, lv; level.

The resulting image is a composite of a grayscale image and a fluorescence image. For fluorescence image processing, the fluorescence signal was extracted exclusively from the composite image, and the grayscale component was subtracted using MATLAB R2020a (MathWorks, Natick, MA, USA). After the removal of the visible light image, the maximum value within the ROI was obtained (Figure 2A).

The laser-assisted laparoscopic NIR camera system offers endoscopic NIR visualization (ENV) modes ranging from 0 to 5, which provide varying fluorescence intensities. The ENV level of the camera determines the contribution of detected FI to the grayscale image, influencing the shape and position of the FI contrast curve. Since quantitative perfusion parameters vary with changes in the ENV level, it is crucial to obtain colon perfusion images using pre-selected ENV levels. To analyze quantitative perfusion parameters at each ENV level, an equation capable of adjusting the ENV level in ICG images was applied for image processing (Figure 2B). In this equation, B denotes the FI, E represents the camera’s ENV level, and k indicates the brightness attenuation value over distance:

$$B\left(i.\;j\right)=255\left(1-\frac{1}{1+{\left(\frac{I_{nir}\left(i,\;j\right)}{k}\right)}^{E}}\right)$$

FI is also significantly influenced by the distance between the camera lens and the surface of the colon. We observed that motion artifacts caused by the surgeon’s hand tremor while holding the camera, as well as movements from the patient’s pulse and respiration, introduced noise in FI by altering this distance. Even when imaging an object with consistent brightness, the received signal is inversely proportional to the square of the distance from the camera. To account for this characteristic, an image processing technique was applied to reduce motion artifacts caused by distance variations (Figure 2C). In this model, d represents the distance between the camera and the subject, while A is a characteristic constant based on the camera’s field of view:

$$k=A\frac{1}{d^{2}}$$

The mean of the integer values within the specified ROI was calculated to reduce errors caused by external factors. To compensate for noise caused by physical factors such as hand tremors and heartbeats, a Gaussian moving average filter was applied to smooth 20 consecutive frames (0.67 s) to obtain a clearer graph pattern (Figure 2C).

### 2.5. Quantitative Perfusion Analysis

**Figure 3:**
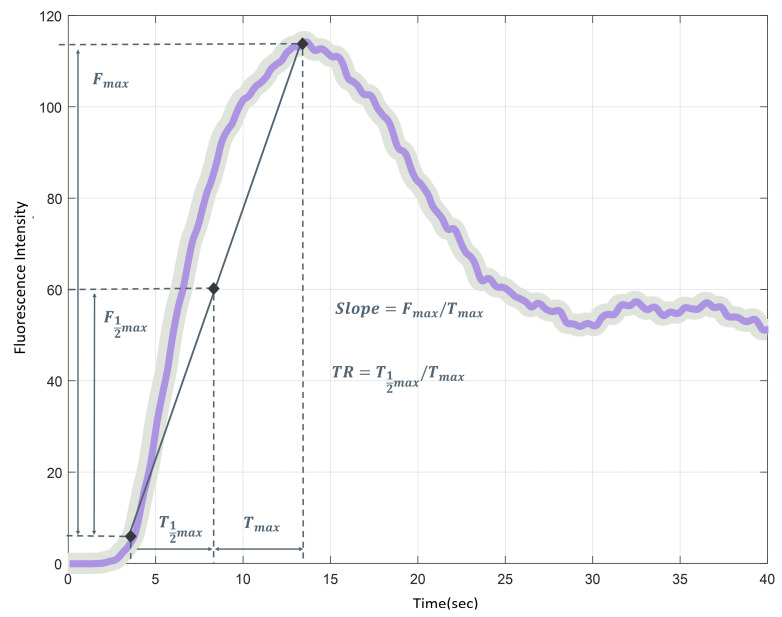
Time-fluorescence intensity (FI) graph for quantitative perfusion analysis. T_1/2MAX_; the time to reach half of the maximum FI, T_MAX_; the time taken to reach maximum FI, perfusion time ratio (TR = T_1/2MAX_/T_MAX_); the ratio of fluorescence expression time, F_1/2MAX_; the half of maximum FI, F_MAX_; the maximum brightness of FI, slope (F_MAX_/T_MAX_); the slope of the rising curve from the initial fluorescence expression to maximum FI.

On the day of surgery, operation recording videos were collected, focusing on the central part of the transverse colon with favorable blood flow conditions. The quantitative perfusion analysis program (ICG Analyzer Program 8.0, developed by the Microprocessor Application Laboratory, Department of Electronic Engineering, Pusan National University, Busan, Korea) was used to analyze perfusion status by generating a curve of time-FI changes and calculating various quantitative perfusion parameters (Figure 3). Additionally, clustering, a machine learning technique, was employed to classify perfusion status as either adequate or inadequate based on distinct patterns. This clustering method could categorize the time-FI change graph patterns over a 40 s period using an artificial intelligence (AI)-based perfusion analysis program developed by the Microprocessor Application Laboratory, Pusan National University, Busan, Korea, as described in a previous publication [21].

We investigated the occurrence of under-saturation and over-saturation of FI with minimal and conventional doses. Under-saturation was defined as a subtle FI change, which was interpreted as unfavorable perfusion because of the absence of a normal FI profile. Conversely, over-saturation was defined as a condition where F_MAX_ reached 250, preventing accurate measurement of the F_MAX_ value. The washout time was defined as the time required for the maximal FI to decrease by 80%. The washout times for the minimal and conventional doses were measured up to 120 s. Analysis accessibility was defined as the successful execution of quantitative analysis on the detailed ROIs using the perfusion analysis program, and these were considered analyzable ROIs. Quantitative perfusion parameters with T_MAX_ of less than 1 s were deemed erroneous and classified as inaccessible for quantitative analysis, thus considered unanalyzable ROIs and excluded from further statistical analysis.

### 2.6. Outcomes

The primary outcome was to determine the minimal dose of ICG that could be used to assess the blood flow status of the colon quantitatively. The suitability of the minimal ICG dose for enabling quantitative perfusion analysis was evaluated based on the following criteria: (1) fluorescence images could be analyzed using the quantitative perfusion analysis program; (2) good blood flow was determined based on acceptable TR and slope values (<0.6 and >4, respectively), as defined in a previous study [9]. If neither condition was met, the minimum ICG dose was considered inappropriate for accurately reflecting good perfusion status.

Secondary outcomes included the evaluation of anastomotic complications, reoperation, and readmission within 30 days post-surgery. Short-term complications, including anastomotic leakage and reoperation, were monitored for 6 weeks after surgery.

### 2.7. Statistics

Continuous variables were analyzed using an independent-sample *T*-test, while categorical variables were evaluated with either the Chi-square test or Fisher’s exact test. Quantitative perfusion parameters were compared between groups using one-way analysis of variance (ANOVA), followed by Tukey’s honestly significant difference test for post-hoc comparisons. For nonparametric analysis, the Kruskal–Wallis test was also performed. A two-way ANOVA was conducted to assess the interaction effects between the groups and individuals, accounting for variations in quantitative perfusion parameters within each group. Outliers from repeated measurements were removed using a general outlier detection method, where an outlier was defined as any value outside 1.5 times the interquartile range (IQR, Q3 − Q1). Specifically, values less than (Q1 − 1.5 × IQR) or greater than (Q3 + 1.5 × IQR) were classified as outliers and excluded from the analysis. The number of outliers identified for each measurement was as follows: TR (*n* = 45), slope (*n* = 8), T_1/2MAX_ (*n* = 5), and T_MAX_ (*n* = 5), while no outliers were found for F_MAX_ and F_1/2MAX_. Statistical analysis was performed using SPSS 28.0 (IBM SPSS, Armonk, NY, USA) and R software (version 4.3.0, R Foundation for Statistical Computing, Vienna, Austria). The significance level for all two-tailed tests was set at *p* < 0.05.

## 3. Results

### 3.1. Patients

The clinicopathological characteristics and clinical outcomes of the patients are shown in Table 2. No adverse effects related to ICG were reported.

### 3.2. Quantitative Perfusion Parameters

**Figure 4:**
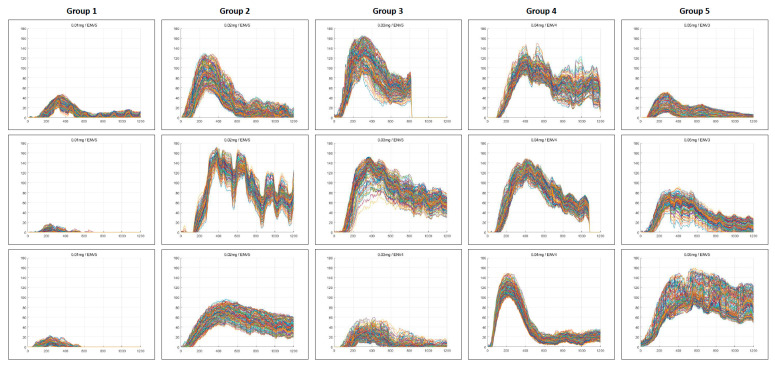
AI-based perfusion pattern assessment. A clustering method utilizing machine learning techniques was employed to categorize perfusion patterns over a 40 s period, facilitated by an AI-based perfusion analysis program. This approach classifies perfusion status as either adequate or inadequate based on identified distinct patterns. Some patterns in Group 1 (86%), Group 3 (26%), and Group 5 (34%) were classified as inappropriate perfusion status. In the time-fluorescence intensity graphs for each patient, the fluorescence intensity graphs for the detailed ROIs were depicted using lines of different colors. AI; artificial intelligence, ENV; endoscopic near-infrared visualization.

A quantitative analysis of colonic perfusion was performed on operation recording videos obtained from 15 patients. The percentage of analysis accessibility was 48.0% in Group 1, which was significantly lower than in Groups 2–5 (84.7–100%) (Table 3). An adequate AI-based perfusion pattern was 14.2% in Group 1, significantly lower than in Groups 2–5 (66.4–100%) (Figure 4). In the conventional dose, all parameters were within the favorable range.

Quantitative perfusion parameters obtained from analyzable ROIs were compared across groups. The mean TR value was within the acceptable range (<0.6) in all groups; however, the mean slope value in Group 1 was 3.7, which fell below the acceptable threshold (>4) and was significantly lower than that of the other groups (*p* < 0.001). F_MAX_ and F_1/2MAX_ were also significantly lower in Group 1 compared with the other groups (Table 4).

A two-way ANOVA was performed to assess the interaction effects between groups and individuals on quantitative perfusion parameters. Statistical differences were observed between individuals within each group. Additionally, when accounting for the interaction between individuals and groups, significant differences in quantitative perfusion parameters were confirmed between the groups (Table 5).

**Figure 5:**
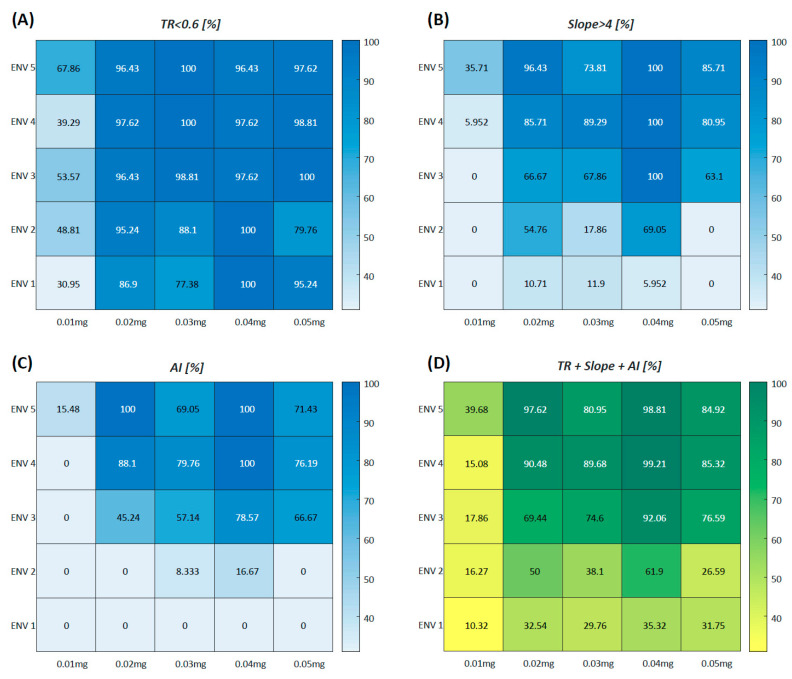
Color map representation of the proportion of successful quantitative perfusion analysis in ENV-transformed image processing. ENV-transformed image processing was conducted, followed by quantitative perfusion analysis across all ENV levels within each group. The proportion of successful analyses within the favorable range was assessed and represented as a blue color map. TR (**A**), slope (**B**), and AI-based perfusion patterns (**C**) were utilized as quantitative perfusion parameters. The average proportion of the three criteria (TR, slope, and AI) is represented as a green color map (**D**). ENV; endoscopic near-infrared visualization, TR; perfusion time ratio, AI; artificial intelligence.

ENV-transformed image processing was conducted, and quantitative perfusion analysis was reperformed across all ENV modes in each group. The proportion of successful analyses within the favorable range was assessed. At ENV levels 3 to 5, the TR (<0.6) ranged from 96.4% to 100%, while the slope (>4) ranged from 63.1% to 100% at doses of 0.02–0.05 mg/kg. In AI-based perfusion pattern analysis, a classification success rate of over 70% was achieved at ENV levels of 4 or higher. The average percentage meeting the three criteria (TR, slope, and AI) exceeded 80% in Groups 2–5 at ENV levels 4 and 5. In contrast, lower ENV levels (ENV ≤ 2) or a minimum ICG dose of 0.01 mg/kg (Group 1) significantly reduced the success rates for quantitative perfusion analysis (Figure 5).

**Figure 6:**
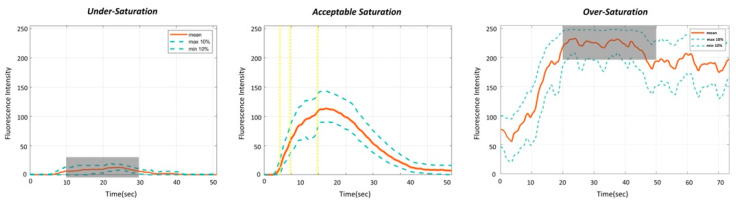
Impact of under-saturation and over-saturation on quantitative analysis. Under-saturation is characterized by a subtle fluorescence intensity (FI) change, resulting in errors in quantitative analysis due to the absence of a normal FI profile. Conversely, over-saturation is defined as a condition where F_MAX_ reaches 250, preventing accurate confirmation of the measured F_MAX_ value.

Under-saturation, which impaired accurate quantitative analysis, occurred in 64.0% of ROIs in Group 1 (0.01 mg/kg). Over-saturation effects were absent with the minimal doses but were present in 20.5% of the ROIs when the conventional dose was used (Figure 6).

When comparing the washout time to reach an 80% reduction in maximal FI, the washout occurred significantly faster at the minimal doses than with the conventional dose (39.0 ± 15.8 s vs. 117.5 ± 4.9 s, respectively, *p* < 0.001). In most cases with the conventional dose, the FI did not decrease by 80% within 2 min of the measurement period.

## 4. Discussion

This study focused on optimizing the minimal dosage of ICG to enhance the rapid washout of fluorescence to improve the efficacy of repeated ICG angiography during colon surgery. We investigated various minimal doses of ICG, ranging from 0.01 to 0.05 mg/kg. The lowest dose (0.01 mg/kg) was considered inappropriate because of unacceptable analysis accessibility, with the slope falling outside the acceptable range. Additionally, the accuracy of quantitative perfusion parameters was significantly lower at ENV levels 1 and 2. These results suggested that a minimal ICG dose of 0.02 to 0.05 mg/kg is sufficient for acceptable quantitative perfusion assessment using a laser-assisted laparoscopic NIR camera system with ENV mode 3 or higher. This proposed dose range is five to ten times lower than the conventional dose (0.2–0.25 mg/kg), potentially allowing for faster washout and enabling immediate repetitive ICG angiography without operation time delays. The rapid clearance of ICG from the bloodstream enhances the flexibility of surgical decision-making by facilitating timely reassessment of perfusion [22].

The ICG angiography is a critical tool for assessing the adequacy of blood flow in the colon, which helps avoid ischemic complications due to insufficient collateral circulation [6,23]. When initial ICG angiography indicates poor perfusion, the colon transection line should be moved proximally and repeated ICG angiography is required to identify a better-vascularized segment of the colon [9,16]. The washout of residual could be influenced by the volume of the ICG solution injected intravenously during the initial test [24]. The conventional ICG dose (0.2–0.25 mg/kg) enables clear fluorescence detection using an NIR camera with a xenon lamp in cases of good perfusion; it requires several minutes for the ICG to be cleared by the liver, delaying any necessary repetition of the ICG angiography [25,26]. The laser-assisted laparoscopic NIR cameras have enhanced fluorescence imaging resolution [24]. These improvements have raised the possibility of using lower doses of ICG, promoting faster washout and enabling immediate repetitive ICG angiography [19,20].

At the conventional dose (0.2 mg/kg), we observed an over-saturation, where the maximum FI exceeded the detectable range of the imaging system in about 20% of ROIs. Over-saturation impedes accurate quantification of maximum FI, leading to distorted parameters such as the slope of the rising FI curve and perfusion time metrics. Moreover, ICG remains in the bloodstream for an extended washout time, delaying its clearance through the liver depending on ICG dosage and hepatic function [22,27]. This results in a gradual decline in FI, making it difficult to perform immediate repetitive tests. Conversely, when the lowest dose (0.01 mg/kg) was evaluated, an underestimation of FI change was observed in 64% of ROIs, a phenomenon known as under-saturation. This resulted in erroneous perfusion parameters, suggesting poor perfusion even when actual blood flow was adequate [28].

This study suggests that an optimal ICG dose for perfusion testing should balance sufficient FI with rapid washout to allow immediate repetitive ICG angiography without compromising diagnostic accuracy. The minimal dose range of 0.02 to 0.05 mg/kg achieves this balance by providing FI for reliable colon perfusion assessment and rapid washout. This optimized minimal dosage is particularly useful in surgeries requiring rapid decision-making, such as determining the precise transection point in colorectal surgery. It facilitates the prompt repetition of the ICG angiography, improving the detection of favorable perfusion segments and reducing the risk of ischemic complications, ultimately enhancing patient outcomes [13,16].

This experimental study has several limitations. First, the primary limitation of this study is the small sample size. In Korea, ICG is not yet approved for clinical use in assessing blood flow during colorectal surgery and is only permitted in IRB-approved studies. During the IRB review process, we were advised to limit enrollment to the minimum patient number required for this experimental study involving drug dosage. Based on this recommendation, we calculated a sample size sufficient to detect significant differences in perfusion parameters. Although only 15 patients were included, the automated perfusion analysis program could analyze 150 ROIs per patient, which was critical for deriving meaningful results. Second, these results are specific to NIR cameras that use lasers as their light source, and the optimal ICG dose may vary for laparoscopic NIR camera systems using xenon lamps or other light sources [17,24]. Additionally, although the reduced minimal ICG dosage enabled adequate quantitative perfusion analysis in this study, it may not provide sufficient FI for subjective assessment by the surgeon’s naked eye viewing the fluorescence on a monitor without quantitative perfusion analysis software. In such cases, a higher ICG dose may still be required. Furthermore, although lowering the ICG dose reduces the total amount of the fluorescent agent administered, it does not necessarily reduce the risk of serious anaphylactic reactions associated with sodium iodide in the ICG solution, which can occur regardless of the infusion dosage [29].

The diagnostic limitations of quantitative perfusion parameters used in this study should also be considered. We utilized T_MAX,_ T_1/2MAX_, TR, and slope, along with an AI-based perfusion pattern; however, each parameter may not fully represent the perfusion status, as the shape of the FI graph is influenced by various external factors [24]. It is important to emphasize that the perfusion time ratio as TR could remain within an acceptable range even when tissue oxygenation is insufficient [30]. So, it is essential to assess multiple parameters, including T_MAX_, T_1/2MAX_, TR, slope, and AI-based perfusion patterns, to facilitate a comprehensive perfusion assessment by the surgeon. Furthermore, the distance between the camera and the colon could affect the FI level. The intravenous infusion rate of ICG could also influence the FI changes, which might vary depending on the anesthesiologist administrating the ICG solution; therefore, in fluorescence imaging studies reflecting FI changes, it is essential to account for various external environmental factors [26].

Based on previous studies [9,17,24], a standardized ICG angiography protocol was established and implemented in this study. The camera was positioned at a constant distance of 5 cm and held as steadily as possible by hand for 2 min. The operating room lights were turned off, and the surgical field was temporarily shielded to minimize interference from the closed-circuit television camera of the operating room. The ICG injection was administered within 10 s, followed by an immediate injection of 20 mL of normal saline to ensure complete delivery of any remaining ICG solution in the fluid line. To standardize ICG angiography for future study, specific recommendations will be needed through careful expert consensus on adjusting factors that influence FI, such as ICG dose, intravenous infusion rate, camera distance, operating room lighting, and other relevant variables.

A new NIR camera system is currently under development, which is expected to improve fluorescence resolution and provide constant FI on the variations in camera distance. Further experiments using more accurate and diverse perfusion parameters with the enhanced laparoscopic NIR camera system are anticipated to facilitate the development of an optimal ICG angiography protocol, enabling the acquisition of more precise fluorescence images and reliable quantitative perfusion analysis.

## 5. Conclusions

This study supports the use of minimal ICG dosage, ranging from 0.02 to 0.05 mg/kg, to optimize repetitive ICG angiography using laser-assisted laparoscopic NIR cameras with level 3 or higher ENV modes. This minimal ICG dose strategy can achieve a balance between providing sufficient FI for acceptable quantitative perfusion assessment and allowing for rapid washout, which facilitates immediate repetition of the ICG angiography.

## Figures and Tables

**Table 1:** Patient group assignment according to minimal ICG dosage (*n* = 15).

	*n*	Initial Dose	Second Dose	Total Dose
Group 1	3	0.01 mg/kg	0.2 mg/kg	0.21 mg/kg
Group 2	3	0.02 mg/kg	0.2 mg/kg	0.22 mg/kg
Group 3	3	0.03 mg/kg	0.2 mg/kg	0.23 mg/kg
Group 4	3	0.04 mg/kg	0.2 mg/kg	0.24 mg/kg
Group 5	3	0.05 mg/kg	0.2 mg/kg	0.25 mg/kg

**Table 2:** Patient characteristics and clinical outcomes (*n* = 15).

Clinical Variables		*n* (%)
Age (yr)	mean ± SD	67.1 ± 11.7
Sex	male	11 (73.3)
	female	4 (26.7)
BMI (kg/m^2^)	mean ± SD	24.1 ± 3.5
ASA score	1	5 (33.3)
	2	10 (66.7)
Hypertension	positive	7 (46.7)
Diabetes mellitus	positive	1 (6.7)
Smoking	positive	3 (20.0)
Cardiovascular disease	positive	2 (13.3)
Chronic liver disease	positive	0
Pathologic stage	I	7 (46.6)
	II	4 (26.7)
	III	4 (26.7)
Anastomotic complication	positive	0
Reoperation	positive	0
Ileus	positive	1 (6.7)

BMI; body mass index, ASA; American Society of Anesthesiologists, SD; standard deviation.

**Table 3:** Accessibility rates of quantitative perfusion analysis on the minimal ICG dosage.

Variables	Group 1 (*n* = 450)	Group 2 (*n* = 450)	Group 3 (*n* = 450)	Group 4 (*n* = 450)	Group 5 (*n* = 450)	*p*-Value
Analysis accessibility	48.00 ± 21.40 ^A^	100.00 ± 0.00 ^B^	88.00 ± 16.41 ^B^	100.00 ± 0.00 ^B^	84.67 ± 17.70 ^C^	0.007
TR (<0.6)	43.53 ± 17.28 ^A^	91.57 ± 12.88 ^B^	87.10 ± 17.40 ^B^	100.00 ± 0.00 ^B^	83.80 ± 19.12 ^C^	0.008
Slope (>4.0)	23.10 ± 40.01 ^A^	99.10 ± 1.56 ^B^	77.57 ± 34.37 ^C^	100.00 ± 0.00 ^B^	75.43 ± 27.97 ^C^	0.032
AI-perfusion pattern	14.23 ± 24.66 ^A^	97.10 ± 5.02 ^B^	74.00 ± 45.03 ^C^	100.00 ± 0.00 ^B^	66.43 ± 35.49 ^C^	0.023

Values are presented as mean ± standard deviation (%). Superscripts (A, B, C) indicate results of the post-hoc test (Tukey’s honestly significant difference): different letters denote significant differences between groups, while the same letters indicate no significant difference. TR; perfusion time ratio, AI; artificial intelligence.

**Table 4:** One-way ANOVA analysis for quantitative perfusion parameters of analyzable regions of interest (ROIs).

Variables	Group 1	Group 2	Group 3	Group 4	Group 5	*p*-Value
	(*n* = 216)	(*n* = 450)	(*n* = 396)	(*n* = 450)	(*n* = 381)	
TR	0.43 ± 0.11 ^A^ [0.35; 0.51]	0.45 ± 0.09 ^B^ [0.39; 0.50]	0.35 ± 0.08 ^C^ [0.30; 0.39]	0.38 ± 0.07 ^D^ [0.32; 0.43]	0.38 ± 0.07 ^D^ [0.33; 0.44]	<0.001
Slope	3.70 ± 2.17 ^A^ [1.85; 5.57]	13.93 ± 6.51 ^B^ [6.58; 19.92]	13.92 ± 6.33 ^B^ [7.86; 17.87]	17.06 ± 6.01 ^C^ [12.63; 22.51]	7.48 ± 3.08 ^D^ [5.47; 9.18]	<0.001
T_1/2MAX_	2.61 ± 1.00 ^A^ [1.97; 3.14]	3.41 ± 0.97 ^B^ [2.72; 4.05]	2.45 ± 0.64 ^A^ [2.09; 2.75]	2.68 ± 0.98 ^A^ [1.43; 3.57]	3.90 ± 2.19 ^C^ [2.14; 6.27]	<0.001
T_MAX_	5.92 ± 1.35 ^A^ [5.09; 6.27]	7.84 ± 2.79 ^B^ [5.74; 10.34]	7.28 ± 1.36 ^C^ [6.64; 7.76]	7.01 ± 2.03 ^C^ [4.77; 8.91]	9.53 ± 5.14 ^D^ [5.17; 15.02]	<0.001
F_1/2MAX_	10.74 ± 6.61 ^A^ [5.03; 17.26]	47.99 ± 17.22 ^B^ [33.37; 68.21]	49.81 ± 20.97 ^B^ [24.32; 64.78]	54.52 ± 6.64 ^C^ [49.57; 59.72]	35.13 ± 16.27 ^D^ [22.78; 47.41]	<0.001
F_MAX_	21.40 ± 13.24 ^A^ [9.74; 34.35]	95.64 ± 34.23 ^B^ [66.51; 135.91]	98.88 ± 41.59 ^B^ [48.44; 129.09]	108.68 ± 13.20 ^C^ [98.91; 119.25]	70.29 ± 32.59 ^D^ [45.46; 95.59]	<0.001

Quantitative perfusion parameter values are presented as mean ± standard deviation [95% confidence interval]. Superscripts (A, B, C, D) indicate post-hoc test (Tukey’s honestly significant difference) results: different letters denote significant differences between groups, while the same letters indicate no significant difference. TR; perfusion time ratio.

**Table 5:** Two-way ANOVA analysis for quantitative perfusion parameters of analyzable regions of interest (ROIs).

Variables		Group 1 (*n* = 216)	Group 2 (*n* = 450)	Group 3 (*n* = 396)	Group 4 (*n* = 450)	Group 5 (*n* = 381)	Factor	*p*-Value
TR	Ind 1	0.43 ± 0.13	0.54 ± 0.06	0.36 ± 0.10	0.36 ± 0.05	0.39 ± 0.08	Group	<0.001
	Ind 2	0.42 ± 0.09	0.43 ± 0.06	0.36 ± 0.11	0.45 ± 0.05	0.38 ± 0.08	Ind	<0.001
	Ind 3	0.42 ± 0.07	0.38 ± 0.06	0.33 ± 0.04	0.32 ± 0.04	0.37 ± 0.07	Group × Ind	<0.001
Slope	Ind 1	5.53 ± 1.19	20.83 ± 1.9	18.19 ± 3.42	13.16 ± 1.28	10.47 ± 2.69	Group	<0.001
	Ind 2	2.40 ± 0.74	15.04 ± 3.12	4.51 ± 1.65	13.10 ± 1.75	6.03 ± 1.43	Ind	<0.001
	Ind 3	1.25 ± 1.05	5.93 ± 0.92	16.53 ± 2.63	24.93 ± 3.27	5.66 ± 2.31	Group × Ind	<0.001
T_1/2MAX_	Ind 1	2.59 ± 0.81	3.60 ± 0.30	2.37 ± 0.68	3.23 ± 0.50	2.39 ± 0.41	Group	<0.001
	Ind 2	2.27 ± 0.73	2.34 ± 0.60	2.58 ± 0.90	3.42 ± 0.34	6.50 ± 0.80	Ind	<0.001
	Ind 3	3.02 ± 1.41	4.30 ± 0.64	2.44 ± 0.27	1.39 ± 0.09	2.07 ± 0.79	Group × Ind	<0.001
T_MAX_	Ind 1	6.02 ± 0.80	6.71 ± 0.58	6.81 ± 1.12	8.94 ± 0.75	6.42 ± 1.69	Group	<0.001
	Ind 2	5.43 ± 1.11	5.53 ± 1.54	7.69 ± 1.96	7.66 ± 0.89	16.13 ± 1.76	Ind	<0.001
	Ind 3	6.20 ± 2.16	11.28 ± 1.45	7.46 ± 0.85	4.43 ± 0.48	5.28 ± 1.05	Group × Ind	<0.001
F_1/2MAX_	Ind 1	16.57 ± 3.39	70.05 ± 2.94	62.28 ± 7.59	58.71 ± 4.91	31.61 ± 3.38	Group	<0.001
	Ind 2	6.38 ± 1.82	40.61 ± 9.66	16.61 ± 4.75	49.74 ± 5.68	51.62 ± 9.11	Ind	<0.001
	Ind 3	3.21 ± 2.19	33.33 ± 5.57	61.03 ± 7.18	55.11 ± 5.96	14.69 ± 5.87	Group × Ind	<0.001
F_MAX_	Ind 1	33.08 ± 6.74	139.44 ± 6.12	123.16 ± 14.84	117.06 ± 9.86	63.11 ± 6.70	Group	<0.001
	Ind 2	12.66 ± 3.66	80.97 ± 19.15	32.98 ± 9.43	99.33 ± 11.25	103.46 ± 17.84	Ind	<0.001
	Ind 3	6.27 ± 4.27	66.50 ± 11.12	121.58 ± 14.29	109.67 ± 11.91	29.26 ± 11.73	Group × Ind	<0.001

Quantitative perfusion parameter values are presented as mean ± standard deviation. Ind; individual. Group × Ind; the interaction between individuals and groups, TR; perfusion time ratio.

## Data Availability

Data presented in this study are available on request from the corresponding author. These data are not publicly available due to ethical and privacy reasons.

## Abbreviations

NIR	near-infrared
ICG	indocyanine green
FI	fluorescence intensity
ASA	American Society of Anesthesiologists
SMA	superior mesenteric artery
MCA	middle colic artery
ICA	ileocolic artery
ROI	region of interest
RGB	red, green, and blue color channels
ENV	endoscopic near-infrared visualization
AI	artificial intelligence
T_1/2MAX_	time to reach half of the maximum FI
T_MAX_	time taken to reach the maximum FI
TR	perfusion time ratio (T_1/2MAX_/T_MAX_), the ratio of fluorescence expression time
F_1/2MAX_	half of maximum FI
F_MAX_	maximum brightness of FI
Slope	(F_MAX_/T_MAX_), the slope of the rising curve from the initial to maximum FI
ANOVA	analysis of variance
BMI	body mass index
